# Controllable Preparation of Low-Cost Coal Gangue-Based SAPO-5 Molecular Sieve and Its Adsorption Performance for Heavy Metal Ions

**DOI:** 10.3390/nano15050366

**Published:** 2025-02-27

**Authors:** Le Kang, Boyang Xu, Pengfei Li, Kai Wang, Jie Chen, Huiling Du, Qianqian Liu, Li Zhang, Xiaoqing Lian

**Affiliations:** 1 College of Materials Science and Engineering, Xi’an University of Science and Technology, Xi’an 710054, China; xuboyanglwz@163.com (B.X.); lipengfei34543@163.com (P.L.); hldu@xust.edu.cn (H.D.); liuqq@xust.edu.cn (Q.L.); lianxiaoqing@126.com (X.L.); 2 School of Electrical Engineering, Qingdao University, Qingdao 266071, China; wkwj888@163.com; 3 Shaanxi Key Laboratory of Catalytic Materials and Technology, Kaili Catalyst & New Materials Co., Ltd., Xi’an 710054, China; li.zhang@xakaili.com

**Keywords:** coal gangue, SAPO-5 molecular sieve, hydrothermal method, heavy metal ion adsorption, adsorption kinetics

## Abstract

With the advancement of industrial production and urban modernization, pollution from heavy metal ions and the accumulation of solid waste have become critical global environmental challenges. Establishing an effective recycling system for solid waste and removing heavy metals from wastewater is essential. Coal gangue was used in this study as the primary material for the synthesis of a fully coal gangue-based phosphorus-silicon-aluminum (SAPO-5) molecular sieve through a hydrothermal process. The SAPO-5 molecular sieve was characterized through several methods, including X-ray diffraction (XRD), scanning electron microscopy (SEM), BET surface analysis, Fourier-transform infrared (FT-IR) spectroscopy, and X-ray photoelectron spectroscopy (XPS), to examine its mineral phases, microstructure, pore characteristics, and material structure. Adsorption performance towards wastewater with Cd^2+^ and Pb^2+^ ions was investigated. It was found that the adsorption processes of these ions are well described by both the pseudo-second-order model and the Langmuir isotherm. According to the Langmuir model, the coal gangue-based SAPO-5 molecular sieve exhibited maximum adsorption capacities of 93.63 mg·g^−1^ for Cd^2+^ and 157.73 mg·g^−1^ for Pb^2+^. After five cycles, the SAPO-5 molecular sieve retained strong stability in adsorbing Cd^2+^ and Pb^2+^, with residual adsorption capacities of 77.03 mg·g^−1^ for Cd^2+^ and 138.21 mg·g^−1^ for Pb^2+^. The excellent adsorption performance of the fully solid waste coal gangue-based SAPO-5 molecular sieve is mainly attributed to its mesoporous channel effects, the complexation of -OH functional groups, and electrostatic attraction.

## 1. Introduction

Rapid industrialization and urbanization have led to the discharge of significant amounts of wastewater containing heavy metals such as cadmium (Cd), mercury (Hg), lead (Pb), copper (Cu), chromium (Cr), and zinc (Zn) into natural water bodies [1]. Cd, a naturally occurring heavy metal widely used in battery manufacturing, plating, and pigment production, poses significant risks when present in excess. It not only causes soil and water pollution but also accumulates in the food chain, ultimately impacting human health. High levels of Cd can cause a decline in lung function, kidney damage, bone diseases, cardiovascular issues, and potentially death [2]. Pb is a naturally occurring heavy metal that has a wide range of applications in industry and daily life. However, lead and its compounds are highly toxic, particularly lead ions (Pb^2+^). Excess Pb^2+^ in the human body can cause neurological damage, kidney disease, reproductive system issues, and developmental delays in children’s intelligence [3]. Thus, the removal of heavy metal ions from wastewater plays a critical role in public health, industrial operations, everyday life, and ecological restoration. Alnasrawi [3] fabricated layered double hydroxide-based montmorillonite cation clay composites via a co-precipitation technique, achieving 89.1% adsorption efficiency for Pb^2+^ and a corresponding adsorption amount of 132.83 mg·g^−1^. Akale [4] reported that a polysulfone membrane modified with Al_2_SiO_6_ achieved the adsorption percentage of over 95% for Pb^2+^ under a pressure of 2 bar. Benmansour [5] employed ion flotation technology using sodium dodecyl sulfate as a collector, achieving a removal efficiency of over 95% for Cd^2+^ in monometallic solutions. In water treatment, adsorption has emerged as a key technique for removing heavy metal ions, owing to its ease of use, low cost, and wide range of applications. As a result, this study simulated heavy metal pollution in untreated wastewater from the electroplating and smelting industries, and the creation of affordable and readily available adsorbents has emerged as a primary area of focus in ongoing research.

The molecular sieve is composed of a three selectively filter molecules based on pore size and possesses excellent ion exchange and selee-dimensional framework formed by [SiO_4_]^4−^ and [AlO_4_]^5−^ tetrahedra, with an orderly arrangement of pore structures [6,7,8]. It can activate adsorption properties. This makes it highly applicable in the purification of liquid and gaseous pollutants, especially in removing heavy metal ions from wastewater [9]. As a significant byproduct of coal mining, coal gangue (CG) is generated in large quantities with low utilization rates, causing considerable environmental burdens. Its comprehensive utilization has become a key focus of research in the fields of environment and energy [10]. CG is rich in silicates, aluminates, and small amounts of iron oxides, with the highest concentrations of silicon and aluminum, and can be used to prepare molecular sieves. Kang [11] synthesized the 13-X molecular sieve from CG using an alkaline fusion-hydrothermal method without a template agent, demonstrating effective adsorption of Cd^2+^. Wang [12] synthesized an LTA-type molecular sieve based on coal gangue via a hydrothermal method, which demonstrated excellent adsorption performance for Cu^2+^. Although various molecular sieves have been prepared from CG, reports on SAPO-5 molecular sieve with the AFI structure are still lacking. Due to their distinctive pore structure and stability, phosphorus–silicon–aluminum molecular sieves (SAPOs) hold great promise in adsorption processes. In particular, the SAPO-5 molecular sieve features a one-dimensional channel system composed of twelve-membered rings formed by tetragonal and hexagonal rings. Due to its negatively charged framework, SAPO-5 is widely applied in fields such as gas separation, catalysis, and pollutant adsorption [13].

This study used CG as the source of silicon and aluminum, with aluminum isopropoxide providing external aluminum and phosphorus source provided by phosphoric acid. Using a hydrothermal method, an environmentally friendly coal gangue-based SAPO-5 molecular sieve adsorbent was synthesized and its efficiency in removing heavy metals from untreated wastewater in the electroplating and smelting industries was evaluated. The impact of different parameters, including initial concentration, adsorbent dosage, temperature, time, and pH on adsorption performance was explored. Adsorption kinetics, isotherms, and various characterization techniques were employed to investigate the adsorption mechanism of the coal gangue-derived SAPO-5 molecular sieve adsorbent. The findings of this study lay a new theoretical and experimental foundation for utilizing CG waste efficiently and treating heavy metal ion-contaminated wastewater, playing an important role in the sustainable development of the ecological environment.

## 2. Experimental

### 2.1. Materials

The CG used in this study was sourced from the Shendong Coal Mining Group, located in Shaanxi, China. Aluminum isopropoxide (C_9_H_21_AlO_3_, AR, ≥99.0%) was purchased from Yangzhou Suoao New Materials Co., Ltd., located in Yangzhou, China. Phosphoric acid (H_3_PO_4_, AR, ≥85.0%) was acquired from Hebei Pengfa Chemical Co., Ltd., located in Cangzhou, China. Triethylamine (TEA) (C_6_H_15_N, AR, ≥99.9%) was supplied by Shandong Keyuan Biochemical Co., Ltd., located in Hezhe, China. Hydrofluoric acid (HF, AR, ≥40.0%) and hydrochloric acid (HCl, AR, ≥36.0%) were both sourced from Xilong Scientific Co., Ltd., located in Shantou, China. Cadmium nitrate (Cd(NO_3_)_2_·4H_2_O, AR, ≥99.9%), copper nitrate (Cu(NO_3_)_2_·3H_2_O, AR, ≥99.9%) and lead nitrate (Pb(NO_3_)_2_·2H_2_O, AR, ≥99.9%) were obtained from Hubei Chengfeng Chemical Co., Ltd., located in Wuhan, China. All experimental water used was self-prepared deionized water.

### 2.2. Characterization

X-ray fluorescence (XRF) spectroscopy (PANalytical, AxiosmX, Almelo, The Netherlands) was employed for the quantitative analysis of CG’s chemical composition. X-ray diffraction (XRD, Bruker Corporation, Bruker D8 Advance, Marburg, Germany) measurements were performed on the material to determine its crystal structure with a scan rate of 5°/min and a range of 5° to 55°. A field emission scanning electron microscope (FESEM, TESCAN, MIRA4, Brno, Czech Republic) was employed to examine the microstructure and surface morphology of the samples, while energy dispersive spectroscopy (EDS, Xplore, Xplore 30, Amsterdam, The Netherlands) was used to determine the elemental composition. A Micromeritics ASAP 2020 analyzer (Micromeritics, ASAP 2020, Norcross, GA, USA) was employed to determine the specific surface area and pore size distribution of the samples through nitrogen adsorption–desorption isotherms at −196 °C. Prior to testing, the samples were vacuum degassed at 250 °C for 2.5 h. The micropore volume was calculated using the t-Plot method, and the pore size distribution was obtained based on the BJH (Barrett-Joyner-Halenda) method, which is based on capillary condensation theory. An X-ray photoelectron spectrometer (XPS, Thermo Fisher Scientific, Escalab 250xi, Waltham, MA, USA) was employed to investigate the chemical states of the atoms on the material’s surface. A Fourier-transform infrared spectrometer (FT-IR, Thermo Fisher Scientific, Nicolet SummitX NicoletiS10, Waltham, MA, USA) was employed to analyze the structure and composition of the materials. An inductively coupled plasma optical emission spectrometer (ICP-OES, Thermo Fisher Scientific, iCAP 7400, Waltham, MA, USA) was used to determine the concentration of heavy metal ions in the solution before and after adsorption. Zeta potential analyzer (Malvern Panalytical, Zetasizer Nano ZS90, Malvern, Worcestershire, UK) was used to analyze the surface charge properties of the sample.

### 2.3. Synthesis of Coal Gangue-Based SAPO-5 Molecular Sieves

**Figure 1:**
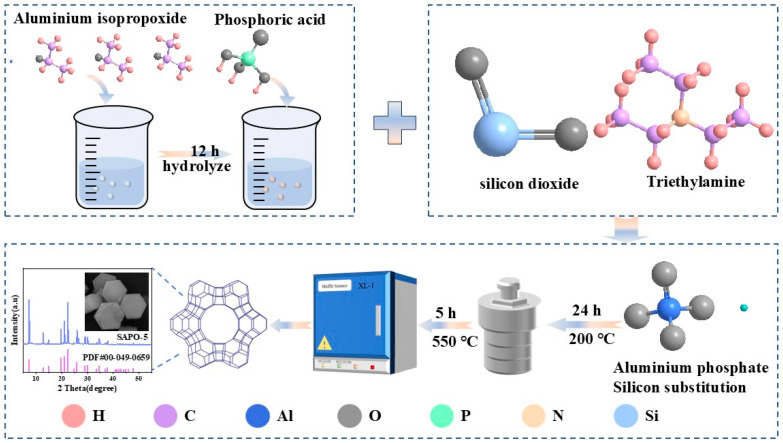
Preparation flowchart of coal gangue-based SAPO-5 molecular sieve.

A calcination pre-treatment of CG is required before the synthesis of the SAPO-5 molecular sieves. This process aims to remove excess carbon and convert the kaolinite present in the CG into an amorphous form of metakaolin through high-temperature calcination. CG is first ground and sieved, then calcined at 800 °C for 6 h in a muffle furnace, resulting in pre-treated coal gangue (PCG) for the preparation of SAPO-5 molecular sieves. Aluminum isopropoxide was pre-hydrolyzed for 12 h, and then H_3_PO_4_, PCG, TEA, and HF were sequentially added according to the molar ratio of 0.3 SiO_2_:Al_2_O_3_:P_2_O_5_:1.5 TEA:0.2 HF:40 H_2_O. Following aging, the mixture underwent hydrothermal synthesis at 200 °C for 24 h in a stainless steel reactor, lined with polytetrafluoroethylene. After natural cooling, the material was washed to a pH of 7, then dried and subjected to calcination at 550 °C for 5 h in a muffle furnace to produce the SAPO-5 molecular sieve. The flowchart of the molecular sieve preparation process is shown in Figure 1.

### 2.4. Adsorption Experiments of Coal Gangue-Based SAPO-5 Molecular Sieve on Cd^2+^ and Pb^2+^ Containing Solutions

The SAPO-5 molecular sieve was washed with deionized water to a pH near 7, and then dried at 70 °C for 5 h before being used to adsorb Cd^2+^ and Pb^2+^. To simulate wastewater containing Cd^2+^ and Pb^2+^, solutions with varying concentrations of Cd(NO_3_)_2_·4H_2_O and Pb(NO_3_)_2_·2H_2_O were prepared. A 100 mL solution was taken, and a specific mass of SAPO-5 molecular sieve was added. For adsorption, the mixture underwent magnetic stirring for 30 to 180 min, after which centrifugation was performed to separate the solution containing the adsorbed substances. The collected supernatant was used to calculate the adsorption amount.

*Q_e_* (mg·g^−1^), representing the adsorption amount, can be calculated using the following general expression [11]:

*Q_e_* = (*C*_0_ − *C_e_*) *V*/*m* (1)

*Q_t_* (mg·g^−1^) at various time intervals is the adsorption amount [11]:

*Q_t_* = (*C*_0_ − *C_t_*) *V*/*m* (2)

The removal rate, denoted as *η*, is typically represented as [11]:

*η* = (*C*_0_ − *C_e_*)/*C*_0_ × 100% (3)

In Equations (1)–(3), *Q_e_* (mg·g^−1^) and *Q_t_* (mg·g^−1^) are used to describe the equilibrium and time-dependent adsorption capacities for Cd^2+^ and Pb^2+^, respectively. In this context, *C*_0_ (mg·L^−1^), *C_t_* (mg·L^−1^), and *C_e_* (mg·L^−1^) refer to the initial concentration, the concentration at time *t*, and the equilibrium concentration of the adsorbate solution, respectively. The volume of the adsorbate solution is represented by *V* (L), *m* (g) indicates the amount of molecular sieve used, and *η* reflects the removal efficiency of heavy metal ions. The removal of heavy metal ions from wastewater by molecular sieves is a type of liquid-solid adsorption process. The pseudo-first-order and pseudo-second-order kinetic models will be used to analyze the adsorption behavior of Cd^2+^ and Pb^2+^ ions on coal gangue-based SAPO-5 molecular sieves, aiming to understand the kinetics of the adsorption process in wastewater containing heavy metal ions. In addition, the adsorption kinetics curves will be fitted and analyzed using these models.

The Lagergren model for pseudo-first-order kinetics is usually written as follows [11]:

*ln* (*Q_e_* − *Q_t_*) = *lnQ_e_* − *K*_1_*t* (4)

Taking the natural logarithm of both sides yields [11]:

*Q_t_* = *Q_e_*(1 − *exp*(−*K*_1_*t*)) (5)

The Lagergren model for pseudo-second-order kinetics is usually written as follows [11]:

*t*/*Q_t_* = *t*/*Q_e_* + 1/*K*_2_*Q_e_*^2^ (6)

In Equations (5) and (6), K_1_ (min^−1^) and K_2_ (g/(mg·min)) are the rate constants for the pseudo-first-order and pseudo-second-order kinetics, respectively.

Adsorption isotherms are a key method for evaluating the characteristics of adsorbents, providing valuable insights into the relationship between the material and the substance being adsorbed. This study utilized the Langmuir and Freundlich models to explore the adsorption characteristics of heavy metal ions in wastewater. The Langmuir model is ideal for characterizing uniform monolayer adsorption, and the Freundlich model is more suitable for heterogeneous liquid-solid adsorption phenomena.

The following expression is commonly used to represent the Langmuir model [11]:

*C_e_*/*Q_e_* = *C_e_*/*Q_m_* + 1/*Q_m_ K_L_* (7)

The following expression is commonly used to represent the Freundlich model [11]:

*ln Q_e_* = (1/*n*) *ln C_e_* + *ln K_F_* (8)

In Equations (7) and (8), *Q_m_* (mg·g^−1^) denotes the maximum amount for adsorption, while K_L_ is the Langmuir model’s equilibrium constant. The Freundlich model’s fitting coefficient is represented by K_F_, and 1/*n* stands for the adsorption index in the same model. When 0.10 < 1/*n* < 0.50, adsorption is considered favorable, while 1/*n* > 2 indicates unfavorable adsorption.

The adsorption performance is primarily influenced by thermodynamic parameters, and the thermodynamic equation can be expressed as follows [11]:

*K_d_* = *Q_e_*/*C_e_* (9)

Δ*G* = −*RTlnK_d_* (10)

*lnKd* = Δ*S*/*R* − Δ*H*/*RT* (11)

In Equations (9)–(11), the adsorption temperature is represented by T(K), while K_d_ denotes the thermodynamic equilibrium constant. The Gibbs free energy is given by ∆*G* (kJ·mol^−1^), R (8.314 J/(mol·K)) represents the ideal gas constant, the entropy is expressed as ∆*S* (J/(mol·K)), and the enthalpy is ∆*H* (kJ·mol^−1^).

## 3. Results and Discussion

### 3.1. Analysis of the Physicochemical Properties of CG Before and After Pretreatment

**Figure 2:**
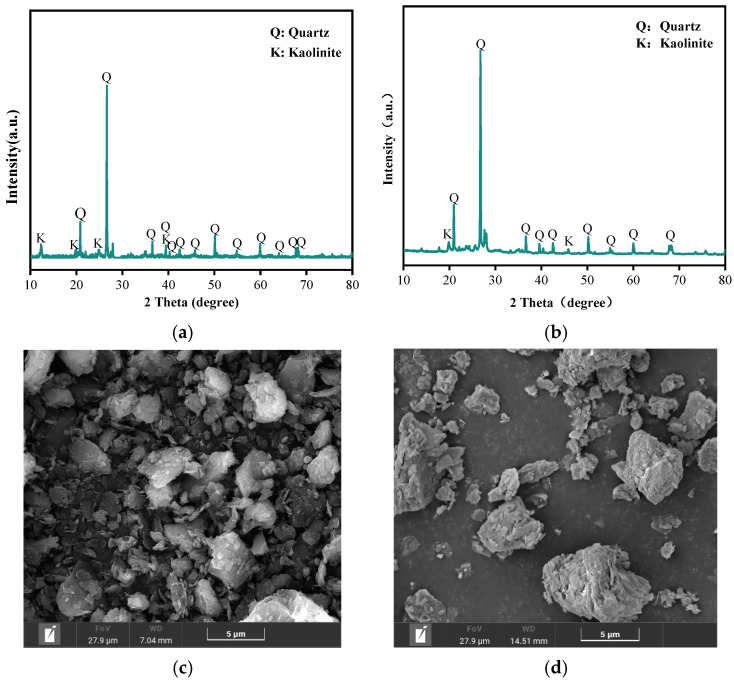
(**a**) XRD pattern of CG; (**b**) XRD pattern of PCG; (**c**) SEM diagram of CG; (**d**) FESEM atlas of PCG.

The CG was ground and sieved, then subjected to calcination at 800 °C for 6 h in a muffle furnace to obtain the PCG. The main chemical components of the CG and the PCG are shown in Table 1, primarily consisting of SiO_2_, Al_2_O_3_, and small amounts of metal oxides. The XRD diffraction patterns of CG and PCG are shown in Figure 2a and Figure 2b, respectively. From the figures, it is evident that quartz and kaolinite are the predominant mineral phases in CG. After calcination pretreatment, the diffraction peak of kaolinite in the PCG is weaker than in the raw sample, suggesting that high-temperature treatment has caused damage to the kaolinite structure due to the loss of structural water. A broad peak appears around 29°, exhibiting characteristics of an amorphous phase [14].

The FESEM images of CG and PCG are shown in Figure 2c,d. The microstructure of the CG is dense, with a prominent flaky structure of kaolinite. The edges of the agglomerated structures are relatively rounded, and the particle size distribution is uneven. Compared to the CG, the PCG exhibits a reduction in the flaky structure, although the layered structure remains present. The particle size is slightly smaller, agglomeration is more pronounced, and the surface is rougher, consistent with the reported transformation of kaolinite to metakaolinite in the literature [15].

### 3.2. Characterization of Coal Gangue-Based SAPO-5 Molecular Sieve

**Figure 3:**
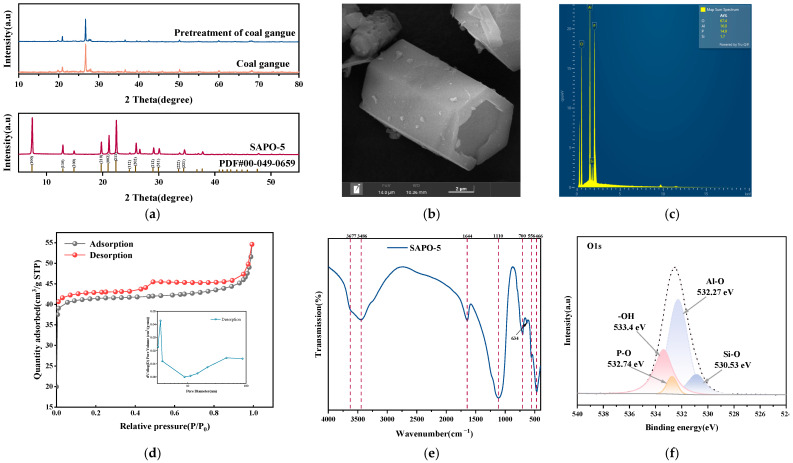
(**a**) XRD spectra of CG, PCG, and SAPO-5 molecular sieve; (**b**) FESEM images of the SAPO-5 molecular sieve; (**c**) EDS spectrum of the SAPO-5 molecular sieve; (**d**) N_2_ adsorption–desorption isotherms and pore size distribution of the SAPO-5 molecular sieve; (**e**) FT-IR spectrum of the SAPO-5 molecular sieve; (**f**) High-resolution spectrum of O1s of the SAPO-5 molecular sieve.

Figure 3a presents the XRD patterns of CG, PCG, and SAPO-5 molecular sieve. Compared to the diffraction peaks of PDF # 00-049-0659 standard card, the synthesized coal gangue-based SAPO-5 molecular sieve exhibits an AFI characteristic structure [16], with distinct and well-defined diffraction peaks at 2θ values of 7.39°, 12.89°, 19.75°, 20.98°, and 22.37° corresponding to the SAPO-5 molecular sieve.

The FESEM images and EDS spectrum of the SAPO-5 molecular sieve are shown in Figure 3b,c. As shown in Figure 3b, the SAPO-5 crystals display clear edges and a regular hexagonal prism structure [17]. According to the EDS data in Figure 3c, the synthesized SAPO-5 molecular sieve contains O, Al, P, and Si in the amounts of 67.4 wt%, 16.0 wt%, 14.9 wt%, and 1.7 wt%, respectively, resulting in a Si-Al molar ratio of about 0.1.

The N_2_ adsorption–desorption isotherms and pore size distribution for the coal gangue-based SAPO-5 molecular sieve are shown in Figure 3d. The adsorption–desorption isotherm indicates that the coal gangue-based SAPO-5 molecular sieve falls under Langmuir type IV, exhibiting an H4-type hysteresis loop. According to the pore size distribution curve and pore size distribution data (Table 2), the average mesopore diameter of the coal gangue-based SAPO-5 molecular sieve is 15.45 nm, indicating a relatively regular mesoporous structure. The good connectivity between the pores allows the adsorbate to move freely within the pores, demonstrating strong adsorption potential [13,18].

As illustrated in Figure 3e, the structural and compositional units in the framework of the coal gangue-based SAPO-5 molecular sieve were analyzed by FT-IR. At 466 cm^−1^, the band corresponds to the bending vibrations associated with the main structural units, which include the silicon–oxygen and aluminumºoxygen tetrahedra. At 556 cm^−1^, the absorption band corresponds to the stretching vibrations of Al-O bonds in the aluminumºoxygen framework. At 634 cm^−1^, the band is assigned to the bending vibrations of P-O bonds within the phosphorus–oxygen framework. The N-H stretching vibrations of amine groups incorporated into the host silica material give rise to the band observed at 700 cm^−1^. At 1100 cm^−1^, the band is associated with the stretching vibrations of Si-O-Si bonds in the silicon–oxygen structure. At 1644 cm^−1^, the bending vibration of hydroxyl groups is observed, and a stretching vibration is detected at 3677 cm^−1^, which is attributed to water adsorption in the crystal channels. The band at 3486 cm^−1^ arises from the hydrogen bonding interactions between Si-OH groups. As shown in the literature [13,19,20,21], the typical adsorption peaks of the coal gangue-based SAPO-5 molecular sieve are observed at similar positions.

Figure 3f presents the high-resolution spectrum of O1s of the coal gangue-based SAPO-5 molecular sieve. In the fitted O1s high-resolution spectrum, the peaks at 530.53 eV, 532.27 eV, 532.74 eV, and 533.4 eV correspond to the silicon–oxygen bonds (Si-O), aluminumºoxygen bonds (Al-O), phosphorus–oxygen bonds (P-O), and surface hydroxyl functional groups (-OH), respectively [19,22,23]. Si-O, Al-O, and P-O are the fundamental components of the SAPO-5 molecular sieve framework, reflecting the covalent bonds formed between silicon, aluminum, phosphorus, and oxygen. Representing the core structural characteristics of the SAPO-5 molecular sieve, these bonds indicate the presence of -OH groups resulting from surface hydroxylation, which suggests a potential for chemical adsorption.

### 3.3. Study of the Adsorption Performance of the SAPO-5 Molecular Sieve for Cd^2+^ and Pb^2+^ and Analysis of Adsorption Behavior

#### 3.3.1. The Effect of Initial Concentration

**Figure 4:**
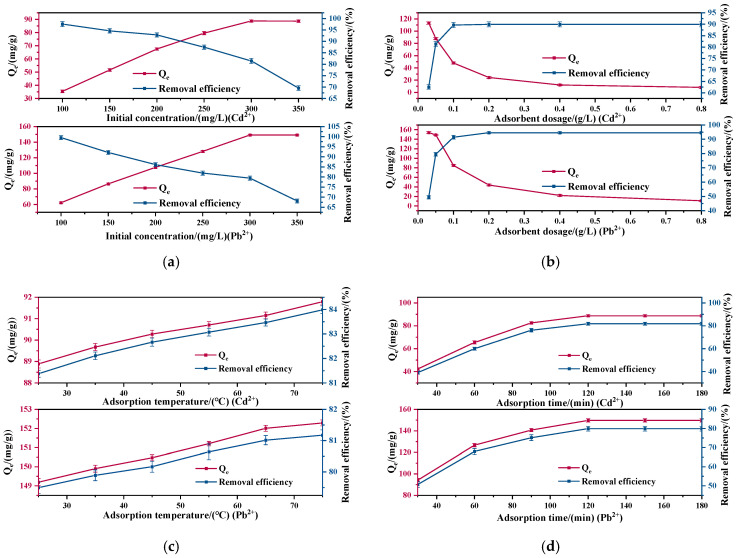
(**a**) The relationship between the initial concentration and the adsorption performance of Cd^2+^ and Pb^2+^ (pH: 6, adsorbent dosage: 0.05 g·L^−1^, adsorption temperature: 25 °C, adsorption time: 120 min); (**b**) the relationship between the adsorbent dosage and the adsorption performance of Cd^2+^ and Pb^2+^ (pH: 6, initial concentration: 300 mg·L^−1^, adsorption temperature: 25 °C, adsorption time: 120 min); (**c**) the relationship between the adsorption temperature and the adsorption performance of Cd^2+^ and Pb^2+^ (pH: 6, initial concentration: 300 mg·L^−1^, adsorbent dosage: 0.05 g·L^−1^, adsorption time: 120 min); (**d**) the relationship between the adsorption time and the adsorption performance of Cd^2+^ and Pb^2+^ (pH: 6, initial concentration: 300 mg·L^−1^, adsorbent dosage: 0.05 g·L^−1^, adsorption temperature: 25 °C).

The impact of initial solution concentration on the adsorption performance of Cd^2+^ and Pb^2+^ is shown in Figure 4a. It can be clearly observed that as the initial concentrations of Cd^2+^ and Pb^2+^ solutions increase, the removal efficiency of the coal gangue-based SAPO-5 molecular sieve for both heavy metal ions gradually decrease. However, the adsorption amount rapidly increases and tends to stabilize. At 300 mg·L^−1^, the initial concentration of the Cd^2+^ and Pb^2+^ solution achieves optimal adsorption performance, attributed to the fixed amount of coal gangue-based SAPO-5 molecular sieve, ensuring a constant number of active adsorption sites on the adsorbent’s surface. As the initial concentration of heavy metal ions in the solution rises, the number of unadsorbed ions remains relatively constant within the specified time frame. An increase in the solution concentration leads to a greater concentration gradient at the adsorbent’s active sites, thus enhancing its adsorption amount per unit mass.

#### 3.3.2. The Effect of Adsorbent Dosage

The effect of adsorbent dosage on the adsorption performance of Cd^2+^ and Pb^2+^ is depicted in Figure 4b. An increase in adsorbent dosage leads to a gradual decrease in the adsorption amount of the coal gangue-based SAPO-5 molecular sieve for both heavy metal ions, accompanied by a rapid rise in removal efficiency, which then levels off. The optimal adsorbent dosage is 0.05 g·L^−1^. When the initial solution concentration is kept constant, the increase in adsorbent dosage results in more adsorption sites and a larger surface area, which consequently improves the removal efficiency. As the total amount of heavy metal ions in a defined volume of solution remains constant, once all the ions are adsorbed, the excess adsorption sites no longer contribute, indicating that saturation has been achieved [24].

#### 3.3.3. The Effect of Adsorption Temperature

The impact of adsorption temperature on the adsorption performance of Cd^2+^ and Pb^2+^ is shown in Figure 4c. It can be observed that as the temperature rises, the ability of the synthesized SAPO-5 molecular sieve to adsorb Cd^2+^ and Pb^2+^ from wastewater increases. Overall, the variation in adsorption amount between 25 °C and 75 °C is relatively small. Raising the temperature accelerates the rate at which the adsorbate diffuses to the surface of the adsorbent, especially in liquid-phase systems. This means that the adsorbate can reach the adsorption sites more quickly, thereby enhancing the adsorption amount. Considering all factors, including economic conditions, the optimal adsorption temperature is 25 °C.

#### 3.3.4. The Effect of Adsorption Time

Figure 4d shows how adsorption time influences the performance of Cd^2+^ and Pb^2+^ adsorption. An increase in adsorption time results in a gradual rise in both adsorption efficiency and amount for Cd^2+^ and Pb^2+^, which then plateau, with 120 min being the optimal adsorption time. With a high number of adsorption sites on the SAPO-5 molecular sieve surface, the adsorption amount increases markedly during the first 30 min. With the continued increase in adsorption time and the progressive occupation of adsorption sites, the rate of amount increase decreases, eventually stabilizing at equilibrium.

#### 3.3.5. The Effect of pH

**Figure 5:**
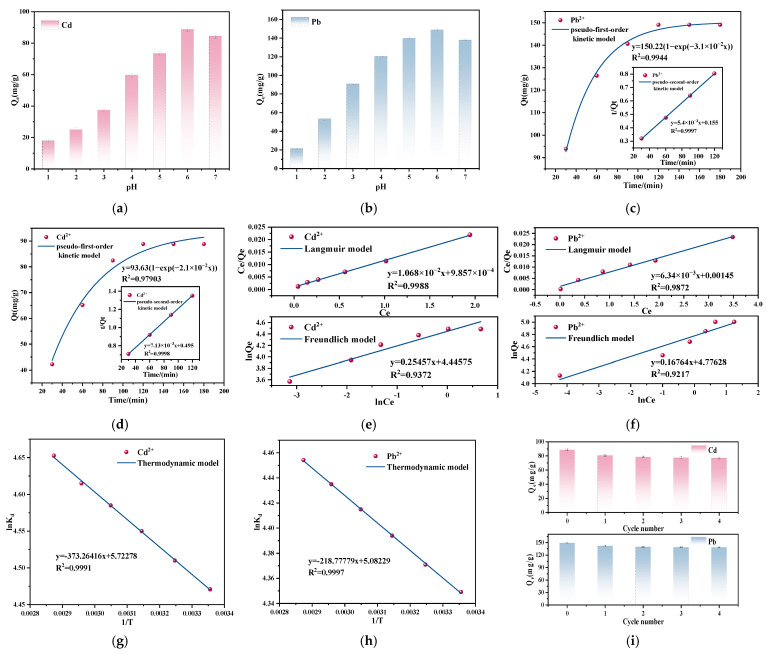
(**a**) The relationship between the pH and the adsorption performance of Cd^2+^ (initial concentration: 300 mg·L^−1^, adsorbent dosage: 0.05 g·L^−1^, adsorption temperature: 25 °C, adsorption time: 120 min); (**b**) the relationship between the pH and the adsorption performance of Pb^2+^ (initial concentration: 300 mg·L^−1^, adsorbent dosage: 0.05 g·L^−1^, adsorption temperature: 25 °C, adsorption time: 120 min); (**c**) kinetic fitting of Pb^2+^ adsorption; (**d**) kinetic fitting of Cd^2+^ adsorption; (**e**) isothermal fitting of Cd^2+^ adsorption; (**f**) isothermal fitting of Pb^2+^ adsorption; (**g**) thermodynamic fitting of Cd^2+^ adsorption; (**h**) thermodynamic fitting of Pb^2+^ adsorption; (**i**) regeneration performance of SAPO-5 molecular sieve in adsorption (pH: 6, initial concentration: 300 mg·L^−1^, dosage of adsorbent: 0.05 g·L^−1^, adsorption temperature: 25 °C, adsorption time: 120 min).

Figure 5a,b present the relationship between solution pH and the adsorption performance of Cd^2+^ and Pb^2+^. With increasing pH, the adsorption amount of the coal gangue-based SAPO-5 molecular sieve for Cd^2+^ and Pb^2+^ increases at first and then decreases, with the optimal pH being 6. At low pH, the surface -OH groups become protonated, resulting in a positively charged surface that causes electrostatic repulsion with Cd^2+^ and Pb^2+^, thus lowering adsorption efficiency. The high concentration of H^+^ in acidic environments competes with heavy metal ions for adsorption sites, which results in fewer effective sites and a consequent decrease in adsorption amount [25]. With an increase in pH, the interaction between H^+^ ions and the functional groups on the surface of the molecular sieve weakens, promoting the adsorption of Cd^2+^ and Pb^2+^ via electrostatic forces. As the pH approaches 7, the formation of Cd(OH)_2_ and Pb(OH)_2_ precipitates, which leads to a decrease in adsorption amount.

The critical role of surface charge in the adsorption process is evident, as the zeta potential of SAPO-5 remains consistently negative across the entire pH range and becomes progressively more negative with increasing pH (Figure S1). At pH 1, the zeta potential is −2.48 mV, and it decreases to −43.8 mV as the pH increases to 7. At pH 6, the surface charge of SAPO-5 is negative with a relatively large absolute value, and the adsorption amount reaches its maximum, indicating that the enhanced surface negative charge favors the adsorption of Cd^2+^ and Pb^2+^. Under lower pH conditions, the surface hydroxyl groups become protonated, resulting in a weaker surface charge. Additionally, the high concentration of hydrogen ions in the solution competes with metal ions for adsorption sites, thereby inhibiting the adsorption of metal ions. At pH 6, the strong negative charge on the surface enhances the electrostatic attraction with positively charged metal ions, thereby significantly improving the adsorption performance [26,27].

#### 3.3.6. Adsorption Kinetics

The results of the adsorption kinetics fitting are shown in Figure 5c,d, and the key parameters for the two kinetic models are listed in Table 3. The pseudo-first-order model is often used to describe the early stages of adsorption, but this usually leads to lower R^2^ values. With R^2^ values of 0.9998 for Cd^2+^ and 0.9997 for Pb^2+^ in the pseudo-second-order model, being closer to 1, more accurately describes the adsorption behavior of the coal gangue-based SAPO-5 molecular sieve. Chemical adsorption, driven by electron sharing or transfer between Cd^2+^, Pb^2+^, and SAPO-5, is the dominant mechanism in this process. A variety of functional groups, including -OH, on the surface are involved in this interaction [28,29].

#### 3.3.7. Adsorption Isotherm

The isotherm fitting curves for Cd^2+^ and Pb^2+^, obtained using the Langmuir and Freundlich models, are shown in Figure 5e,f. The values of Q_m_, K_L_, 1/n, K_F_, and the R^2^ linear correlation coefficient are provided in Table 4.

To analyze adsorption equilibrium, the Freundlich and Langmuir models are commonly applied. The Langmuir model fits for Cd^2+^ (R^2^ = 0.9988) and Pb^2+^ (R^2^ = 0.9872) are both higher than those of the Freundlich model (R^2^ = 0.9372 and R^2^ = 0.9217, respectively), which indicating that the adsorption process is characterized by uniform monolayer adsorption [29,30].

The maximum Q_m_ values for SAPO-5, calculated from the Langmuir model, are 93.63 mg·g^−1^ for Cd^2+^ and 157.73 mg·g^−1^ for Pb^2+^. Table 5 provides a comparison of the Cd^2+^ and Pb^2+^ adsorption capacities of SAPO-5 with various other adsorbents. Our findings demonstrate that SAPO-5 exhibits considerably higher adsorption capacities than the alternatives. The findings indicate that the coal gangue-based SAPO-5 molecular sieve possesses excellent adsorption amount, demonstrating potential applications for capturing heavy metal ions in actual wastewater.

#### 3.3.8. Thermodynamic Study

The results of the thermodynamic study and related parameters are presented in Figure 5g,h, as well as Table 6. Spontaneity of Cd^2+^ and Pb^2+^ adsorption is indicated by the negative ∆G values, which show an increase in magnitude with rising temperature. Higher temperatures contribute to a stronger driving force for adsorption, which accelerates the process. An increase in entropy suggests that the adsorption of Cd^2+^ and Pb^2+^ leads to greater disorder in the system, confirming the attractive interaction between the synthesized SAPO-5 molecular sieve and these ions. The positive enthalpy change indicates that the process of Cd^2+^ and Pb^2+^ adsorption onto the coal gangue-based SAPO-5 molecular sieve is endothermic.

#### 3.3.9. Evaluation of Regeneration Performance of SAPO-5 Molecular Sieve

Desorbing heavy metal ions with 0.1 M HCl restores the SAPO-5 to its original state. As illustrated in Figure 5i, the coal gangue-based SAPO-5 molecular sieve demonstrates a slight decrease in adsorption amount for Cd^2+^ and Pb^2+^ with increasing cycle numbers. After five cycles, the adsorption amount for Cd^2+^ decreased by 5.04%, while that for Pb^2+^ decreased by 7.5%. These results indicate that stable complexes or metal hydroxides formed between the SAPO-5 molecular sieve and heavy metal ions lead to a slight reduction in adsorption amount and make it challenging for complete desorption. Overall, even after five cycles, the SAPO-5 molecular sieve retains a high adsorption amount for Cd^2+^ and Pb^2+^, demonstrating its excellent cyclic adsorption capability.

#### 3.3.10. Competitive Adsorption of Multiple Ions

In wastewater containing heavy metal ions, multiple heavy metal ions are typically present simultaneously. In the competitive adsorption process of Cd^2+^, Pb^2+^, and Cu^2+^ on coal gangue-based SAPO-5 molecular sieve (Figure S2), Pb^2+^ (74.57 mg/g) exhibited a significantly higher adsorption amount compared to Cd^2+^ (54.28 mg/g) and Cu^2+^ (51.70 mg/g). Pb^2+^ has a higher charge density, which allows it to preferentially occupy adsorption sites during the competitive adsorption process. Studies have shown that Pb^2+^ can interact strongly with the functional groups on the surface of the adsorbent through electrostatic interactions and surface complexation reactions. Therefore, in the competitive adsorption of multiple metal ions, Pb^2+^ is typically preferentially adsorbed [42]. In contrast, Cd^2+^ has a lower charge density and smaller ionic radius compared to Pb^2+^, resulting in slightly weaker adsorption amount. However, it still demonstrates strong adsorption potential [43]. Cu^2+^ has the weakest adsorption amount among the three due to its lower charge density, which puts it at a disadvantage in competitive adsorption, although copper ions themselves also possess relatively high adsorption amount [44].

### 3.4. Adsorption Mechanism of SAPO-5 Molecular Sieve

**Figure 6:**
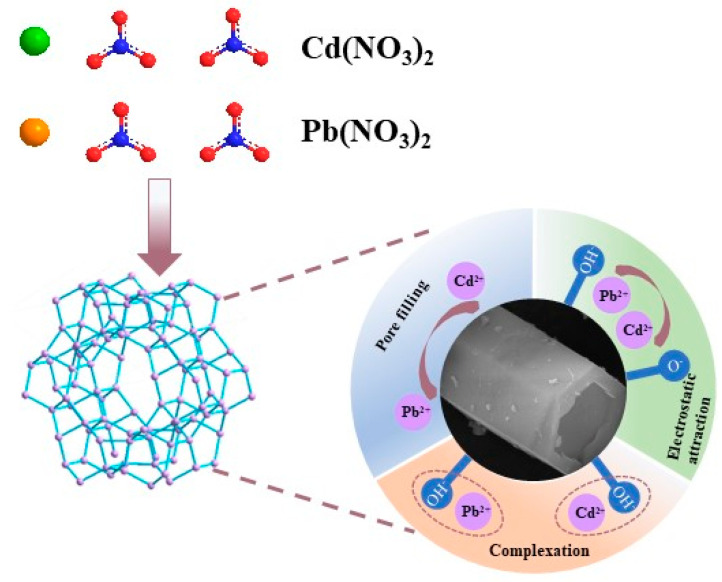
Schematic of the adsorption mechanism of heavy metal ions by coal gangue-based SAPO-5 molecular sieve.

In Section 3.2, the N_2_ adsorption–desorption isotherm analysis indicates an average pore diameter of 2.03 nm for SAPO-5 molecular sieve. The radius of Cd^2+^ is approximately 0.097 nm, while the radius of Pb^2+^ is around 0.102 nm. Given its sufficiently large pore size, SAPO-5 is capable of effectively adsorbing heavy metal ions. The appearance of an H4-type hysteresis loop indicates capillary condensation occurring within the mesopores, suggesting that one of the mechanisms for heavy metal ion adsorption by the SAPO-5 molecular sieve is pore filling. In Section 3.3.5, with a gradual increase in the pH of the solution, the adsorption of Cd^2+^ and Pb^2+^ by SAPO-5 molecular sieve becomes more pronounced, which implies that electrostatic attraction contributes to the mechanism of heavy metal ion adsorption. Figure 6 illustrates the mechanisms involved in the adsorption of heavy metal ions by SAPO-5 molecular sieve based on coal gangue.

**Figure 7:**
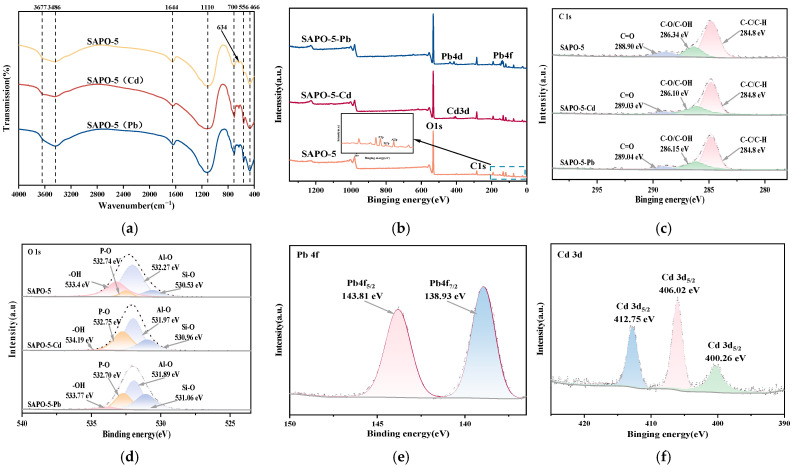
(**a**) FT-IR spectra of SAPO-5, SAPO-5-Cd, and SAPO-5-Pb; (**b**) XPS survey spectra; (**c**) high-resolution spectra of C1s of SAPO-5, SAPO-5-Cd, and SAPO-5-Pb; (**d**) high-resolution spectra of O1s of SAPO-5, SAPO-5-Cd, and SAPO-5-Pb; (**e**) high-resolution spectra of Pb4f of SAPO-5-Pb; (**f**) high-resolution spectra of Cd3d of SAPO-5-Cd.

The FT-IR spectra of SAPO-5 molecular sieve, as well as those obtained after the adsorption of Cd^2+^ (SAPO-5-Cd) and Pb^2+^ (SAPO-5-Pb), are shown in Figure 7a. As shown in the figure, after adsorbing Cd^2+^ and Pb^2+^, the bending and stretching vibration bands of -OH exhibit a slight decrease in intensity and a shift to lower wavenumbers, which suggests their role in the adsorption processes of cadmium and lead [25]. The interaction between oxygen-containing functional groups and Cd^2+^ and Pb^2+^, as shown by the results, implies that complexation is involved in the adsorption process. Figure 7d shows the high-resolution spectra of O1s of SAPO-5, SAPO-5-Cd, and SAPO-5-Pb. As compared to the SAPO-5 molecular sieve, the binding energies of -OH in the ion-adsorbed samples are greater, and the peak areas of -OH have decreased to varying degrees. This indicates that -OH is involved in the complexation of Cd^2+^ and Pb^2+^. Additionally, the Pb4f XPS spectrum (Figure 7e) shows peaks at 138.93 eV (Pb4f_7/2_) and 143.81 eV (Pb4f_5/2_), which are attributed to Pb(OH)_2_ and Pb-O, respectively. The Cd3d XPS spectrum (Figure 7f) reveals peaks at 400.26 eV (Cd3d_5/2_) and 406.02 eV (Cd3d_3/2_), which are attributed to Cd(OH)_2_ and Cd-O, respectively. This further indicates that Cd^2+^ and Pb^2+^ are adsorbed onto the SAPO-5 molecular sieve in a deprotonated form through interactions with hydroxyl groups. Previous research supports this finding, indicating that -OH groups play a role in the adsorption of Cd^2+^ and Pb^2+^ through surface complexation [36].

## 4. Conclusions

The study synthesizes a SAPO-5 molecular sieve from coal gangue, a waste material, using a hydrothermal method for Cd^2+^ and Pb^2+^ removal. The best adsorption performance was achieved with adsorbent dosage of 0.05 g·L^−1^, adsorption temperature of 25 °C, adsorption time of 120 min, pH of 6, and initial concentration of 300 mg·L^−1^. SAPO-5’s uniform monolayer chemical adsorption process is well represented by the pseudo-second-order kinetic model and the Langmuir adsorption isotherm. The maximum equilibrium adsorption amount for Cd^2+^ is 93.63 mg·g^−1^, while for Pb^2+^ it is 157.73 mg·g^−1^. The adsorption mechanisms are primarily attributed to the mesoporous channel effects, complexation of -OH functional groups, and electrostatic attraction. Compared to other adsorbents, the SAPO-5 molecular sieve demonstrates superior adsorption performance for Cd^2+^ and Pb^2+^. The synthesized SAPO-5 molecular sieve shows great promise in removing Cd^2+^ and Pb^2+^ from water, highlighting its potential as a sustainable adsorbent for practical use.

## Figures and Tables

**Table 1:** Chemical compositions of CG and PCG.

Compositions	SiO_2_	Al_2_O_3_	MgO	Na_2_O	K_2_O	CaO	TiO_2_	Fe_2_O_3_	LOI
CG (wt%)	64.58	22.01	0.06	1.01	3.29	1.47	5.58	1.15	0.75
PCG (wt%)	64.50	18.96	1.65	1.70	4.21	0.92	1.15	5.89	0.46

**Table 2:** Pore size distribution of coal gangue and SAPO-5 molecular sieve.

Sample	S_BET_/(m^2^·g^−1^)	Total Pore Volume/(cm^3^·g^−1^)	Microporous Pore Volume/(cm^3^·g^−1^)	Average Mesoporous Pore Size/(nm)
CG	2.04	--	0.01	--
SAPO-5	166.45	0.08447	0.60417	15.4452

**Table 3:** Kinetic model parameters for adsorption of Cd^2+^ and Pb^2+^ onto SAPO-5 sample.

	Pseudo-First-Order Model			Pseudo-Second-Order Model		
	K_1_/min^−1^	Q_e_/(mg·g^−1^)	R^2^	K_2_/(g·mg^−1^·min^−1^)	Q_e_/(mg·g^−1^)	R^2^
Cd^2+^	0.021	93.63	0.979	0.0001027	140.25	0.9998
Pd^2+^	0.031	150.22	0.9944	0.0001881	185.18	0.9997

**Table 4:** The Freundlich and Langmuir isotherm models for the adsorption of Cd^2+^ and Pb^2+^ onto SAPO-5.

	Langmuir			Freundlich		
	K_L_/(L·mg^−1^)	Q_m_/(mg·g^−1^)	R^2^	K_F_/((mg·g^−1^)·(L·mg^−1^)1/n)	1/n	R^2^
Cd^2+^	10.84	93.63	0.9988	85.62	0.25457	0.9372
Pb^2+^	4.37	157.73	0.9872	118.66	0.16764	0.9217

**Table 5:** Comparison of adsorption capacities of adsorbents for Cd^2+^ and Pb^2+^.

Sample	Initial Concentration (mg·g^−1^)	pH	Adsorption Time (h)	Cd^2+^ Adsorption/(mg·g^−1^)	Pd^2+^ Adsorption/(mg·g^−1^)	Refs.
SAPO-5	300	6	2	93.63	157.73	This work
Caltrop shell derived biochar and gelatin/alginate	50	7	4	86.25	--	[25]
NaX zeolite	100	5	2	100.11	--	[28]
Recombinant human ferritin- Synthetic phytochelatin	100	7	20	1.306 (monomer)	--	[31]
Resorcinol-based hyper-cross-linked polymer (R-HCP)	50	10	0.5	10	--	[32]
Graphene oxide/Poly-lactic acid	10	6	4	1.26	--	[33]
Urea-Functionalized mesoporous silica SBA-15	25	5 (Cd^2+^); 4 (Pb^2+^)	2 (Cd^2+^); 1.5 (Pb^2+^)	30.53	43.85	[34]
Magnetic biochars derived from waste marine macro-algal biomass	1200	7	24	23.16	--	[35]
Aged biochar	50	6	6	52.9	74.4	[36]
Crushed concrete fines	40	6	2	--	37	[37]
Carbonized hemp seeds/Fe_3_O_4_ nanoparticles	80	8	2	42.12	--	[38]
Biochar-Zero-Valent-Iron	50	5	24	32.55	--	[39]
f β-cyclodextrin bonded Fe_3_O_4_@SiO_2_	250	6.5	0.75	159.33	160	[40]
Coffee peel-activated charcoal	60	4	24	--	75	[41]

**Table 6:** Thermodynamic parameters of Cd^2+^ and Pb^2+^ at different temperatures.

	T/(K)	ΔG/(kJ·mol^−1^)	ΔH/(kJ·mol^−1^)	ΔS/(J·(mol·K)^−1^)
Cd^2+^	298	−22.7764	3.11	47.58
	308	−23.6601		
	318	−24.5271		
	328	−25.3766		
	338	−26.2324		
	348	−27.1239		
Pb^2+^	298	−23.9899	1.82	42.25
	308	−24.8546		
	318	−25.7114		
	328	−26.5917		
	338	−27.4775		
	348	−28.3189		

## Data Availability

We promise to provide all testing and experimental data as requested at any time.

## Supplementary Materials

The following supporting information can be downloaded at: https://www.mdpi.com/article/10.3390/nano15050366/s1, Figure S1: Zeta potential value of SAPO-5 at pH 1–7. Figure S2: The competitive adsorption of Cd^2+^, Pb^2+^, and Cu^2+^ on the SAPO-5 molecular sieve (initial concentration: 300 mg·L^−1^, adsorbent dosage: 0.05 g·L^−1^, adsorption temperature: 25 °C, adsorption time: 120 min).
