# Supplementary material for: Controllable Preparation of Low-Cost Coal Gangue-Based SAPO-5 Molecular Sieve and Its Adsorption Performance for Heavy Metal Ions
Source: Nanomaterials (Basel). 2025 Feb 27;15(5):366. doi: 10.3390/nano15050366 (PMC11901519; doi:10.3390/nano15050366)
Supplement: Supplementary file 1 [file nanomaterials-15-00366-s001.zip › nanomaterials-3407711-supplementary.pdf]

Controllable Preparation of Low-Cost Coal Gangue-Based SAPO-5 Molecular Sieve and Its Adsorption Performance for Heavy Metal Ions

Le Kang[1,*], Boyang Xu[1], Pengfei Li[1], Kai Wang[2], Jie Chen[1,*], Huiling Du[1], Qianqian Liu[1], Li Zhang[3], Xiaoqing Lian[1]

1 College of Materials Science and Engineering, Xi'an University of Science and Technology, Xi'an, 710054, China

2 School of Electrical Engineering, Qingdao University, Qingdao 266071, China

3. Shaanxi Key Laboratory of Catalytic Materials and Technology, Kaili Catalyst & New Materials Co., Ltd., Xi'an, 710054, China

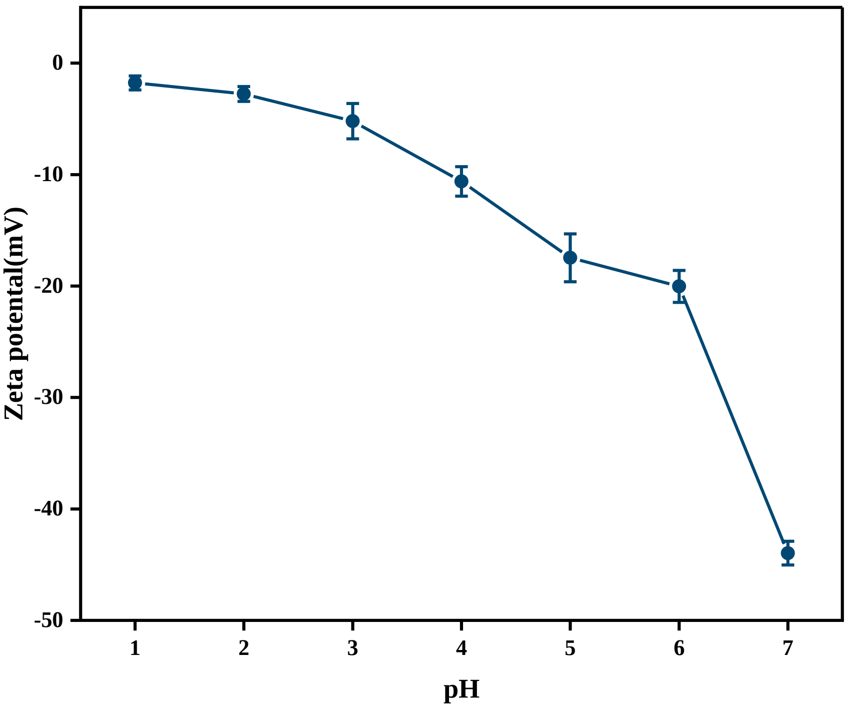

Figure S1 Zeta potential value of SAPO-5 at pH 1-7

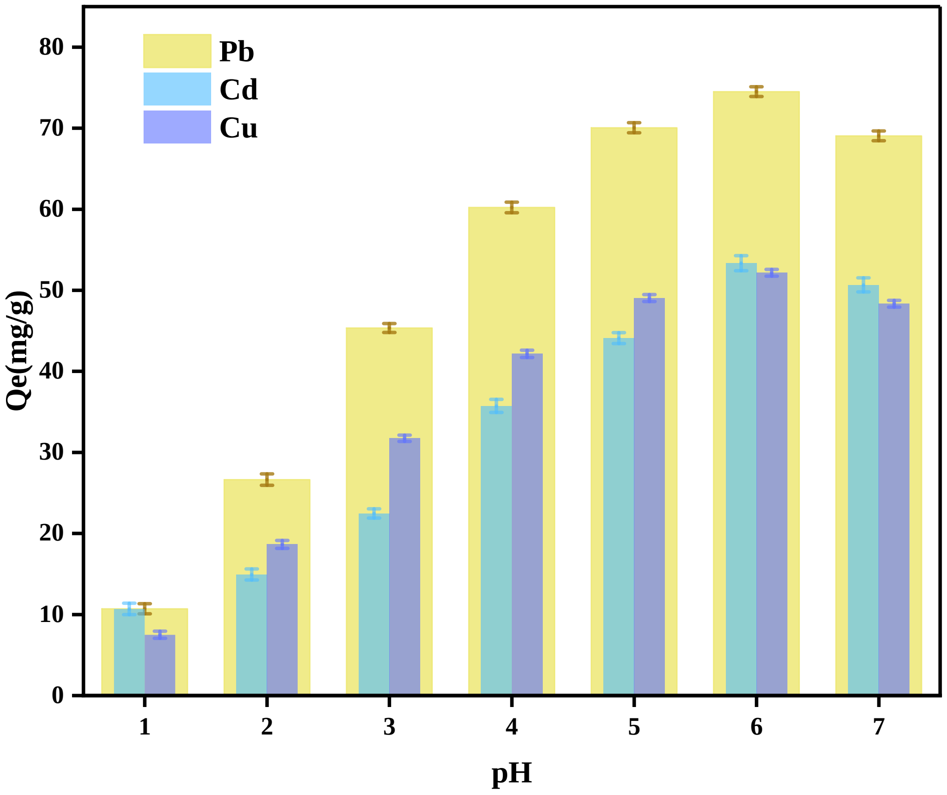

Figure S2 The competitive adsorption of Cd^{2+}, Pb^{2+}, and Cu^{2+} on the SAPO-5 molecular sieve (initial concentration: 300 mg·L^{-1}, adsorbent dosage: 0.05 g·L^{-1}, adsorption temperature: 25 °C, adsorption time: 120 min)
